# Prevalence of physical comorbidities and multimorbidity in Borderline Personality Disorder: risk factors and comparisons with schizophrenia and healthy controls

**DOI:** 10.3389/fpsyt.2026.1726738

**Published:** 2026-09-03

**Authors:** Manoel Dias Ribeiro Junior, Charles-Édouard Giguère, Lionel Cailhol

**Affiliations:** 1 Department of Psychiatry and Addiction, University of Montréal, QC, Montreal, QC, Canada; 2 Centre de recherche de l’Institut universitaire en santé mentale de Montréal, Montreal, QC, Canada; 3 CERVO Brain Research Centre, Université Laval, Montreal, QC, Canada

**Keywords:** Borderline Personality Disorder, chronic illness, multimorbidity, risk factors, schizophrenia

## Abstract

**Background:**

Borderline personality disorder (BPD) is linked to a shorter life expectancy and poorer psychosocial functioning than seen in the general population, with outcomes comparable to those seen in schizophrenia. This is partly explained by the high prevalence of chronic physical illnesses.

**Methods:**

This study aimed to compare rates of multimorbidity and chronic illnesses in individuals with BPD, schizophrenia, and healthy controls, and to examine behavioral risk factors, such as impulsivity, sleep disturbances, and substance use, that may underlie physical health outcomes. We analyzed data from 590 adults enrolled in the Signature database, including validated health and behavioral measures.

**Results:**

Participants with BPD showed the highest rates of multimorbidity (49.4%) and were over four times more likely to report chronic illnesses than controls. Key risk factors associated with increased chronic disease burden included longer sleep latency, higher impulsivity, and smoking, while being a non-smoker was protective.

**Conclusion:**

These findings indicate that physical comorbidities and multimorbidity are at least as prevalent in individuals with BPD as in those with schizophrenia, with age and impulsivity emerging as key contributing factors. While the results underscore the potential value of integrating lifestyle medicine into clinical care, particularly by targeting sleep and substance use, further research is needed to elucidate the causal mechanisms underlying the interplay between BPD and multimorbidity.

## 1 Introduction

Individuals with severe mental illnesses, such as Borderline Personality Disorder (BPD) and schizophrenia, experience significantly poorer physical health outcomes, including higher rates of premature death, chronic diseases, and functional impairment (1, 2). Physical multimorbidity, defined as the co-occurrence of two or more chronic conditions (3), has emerged as a key measure of disease burden in this context. While BPD is primarily characterized by emotional and relational instability, impulsivity, and identity disturbance (4), growing evidence shows that individuals with BPD also face a marked reduction in life expectancy, with excess mortality from both suicide and physical illness (5, 6). Despite this, research has largely focused on psychiatric outcomes, neglecting the physical health dimension.

Contributing factors to this mortality gap include altered stress regulation due to childhood adversity and HPA-axis dysregulation (7, 8), along with behavioral risks such as impulsivity, substance use, and physical inactivity (9). A similar pattern is observed in schizophrenia, where shared mechanisms such as medication side effects, stigma, and health service disparities contribute to a life expectancy reduction of 12–15 years (10, 11). While both disorders exhibit overlapping vulnerabilities, such as impulsivity and emotional dysregulation, comparative studies on physical health outcomes are lacking.

To date, no study has directly compared the prevalence of multimorbidity and its behavioral risk factors among individuals with BPD, schizophrenia, and healthy controls. This gap limits our understanding of shared and distinct pathways to chronic disease in these populations. The current study aims to address this by systematically examining multimorbidity rates and related risk factors (e.g., sleep disturbance, impulsivity, alcohol and tobacco use) across these groups. By identifying key contributors to chronic illness, the findings may inform targeted prevention strategies and promote more integrated models of care for individuals with severe mental illness.

## 2 Materials and methods

### 2.1 Sample description

The sample comprises participants drawn from the Signature Biobank (12). The group with BPD consisted of 158 participants, the schizophrenia group of 283 participants, and the healthy control group of 149 participants.

The Signature database is derived from the *Institut universitaire en santé mentale de Montréal* (IUSMM). Established in 2012, Signature gathers data from patients admitted to the hospital’s psychiatric emergency room between 2012 and 2020 and evaluates them prospectively using 18 psychosocial questionnaires and 23 biomarkers. A control group was recruited from the local community surrounding the hospital and matched for sociodemographic factors. To be included in the control group, individuals must not have visited the psychiatric emergency department within the past 5 years.

The study was approved by the Ethics Committee of CIUSSS de l’Est-de-l’Île-de-Montréal. Project was registered under 2025–3947 on the Nagano-CEMTL.

### 2.2 Measures

#### 2.2.1 BPD and schizophrenia

Psychiatrists evaluated patients upon admission to the psychiatric emergency department (T1), defined as patients who visited the IUSMM psychiatric emergency services between 2012 and 2020. They established preliminary diagnoses of BPD or schizophrenia based on clinical interviews, medical records, and longitudinal history, supplemented by collateral information when available, coding the diagnosis according to the World Health Organization International Classification of Diseases (ICD-10). No structured diagnostic instrument was formally administered. These diagnoses could be reassessed at the end of hospitalization (T2), which refers to the patient follow-up at hospital discharge if it occurred at least 48 hours after being hospitalized, allowing for diagnostic refinement. For the purposes of this study, we used the diagnoses confirmed at T2, as they reflect a more accurate clinical assessment.

The following questionnaires were used from the Signature database:

#### 2.2.2 ESCC-2008 Chronic Conditions V1 (Canadian Community Health Survey - CCHS)

Chronic Conditions V1 (ESCC-2008) (13) is a questionnaire that addresses chronic conditions lasting six months or more and diagnosed by a health care professional. It has been used in some studies to evaluate multimorbidity in the Canadian population (14, 15). It includes 19 questions about the following conditions, to which participants respond with yes or no: asthma, arthritis, back pain, hypertension, migraine, chronic bronchitis, diabetes, heart disease, cancer, ulcers of the intestines or stomach, stroke, urinary incontinence, intestinal disorders (Crohn’s disease, ulcerative colitis, irritable bowel syndrome, fecal incontinence), Alzheimer’s disease or dementia, mood disorders, anxiety, and epilepsy. Psychiatric disorders were not included in the multimorbidity count as the aim of the study was to examine physical comorbidities specifically. In addition, including mood and anxiety disorders could have overlapped with the psychiatric diagnoses defining group membership and confounded comparisons of physical disease burden across groups. We calculated the total of the health problem indicators, resulting in a score that ranges from 0 to 17.

#### 2.2.3 Alcohol Use Disorder: AUDIT-10

The Alcohol Use Disorder Identification Test (AUDIT-10) (16, 17) is a test developed by the World Health Organization to evaluate an individual’s alcohol consumption. It consists of ten self-reported items, each rated on a scale of 0 to 4. The highest possible score is 40, with a score of 8 considered the threshold for identifying potential alcohol-related issues. For this study, we used the total score. The questionnaire’s psychometric properties are satisfactory, with a Cronbach’s alpha and test-retest reliability approximately at 0.8 (18, 19).

#### 2.2.4 Sleep: SHQ

The Sleep Habits Questionnaire (SHQ) (20) is a questionnaire that includes open-ended questions about various sleep habits, such as bedtime and wake-up times during weekdays and weekends, sleep latency, sleep quality, and the frequency and duration of nocturnal awakenings. For this study, we focused on sleep onset latency and awake time.

#### 2.2.5 Impulsivity: UPPS-P

The Impulsive Behavior Scale (UPPS-P) (21) is a tool consisting of 20 items rated on a four-point Likert scale (1): strongly disagree, (2) somewhat disagree, (3) somewhat agree, and (4) strongly agree. The UPPS scale includes four factors: urgency, which refers to the ability to control urges and delay gratification; premeditation, which denotes the tendency to think and plan an action; perseverance, characterized by the tendency to remain focused on a task; and sensation-seeking, defined as the tendency to seek out exciting activities (22). For this study, we examined the five scales derived from the sums of the four items related to each factor: negative urgency, positive urgency, sensation-seeking, lack of perseverance, and absence of premeditation. Each scale has a range from 4 to 16. The Cronbach’s alpha for the questionnaire ranges from 0.70 to 0.84, while test-retest reliability varies from 0.84 to 0.92. This instrument has been validated in French (23).

#### 2.2.6 Tobacco: TABAC

The Tobacco Questionnaire (13) is a four-question evaluation designed to assess tobacco use. It includes questions about the age of onset, current consumption, vaping (e-cigarette) use, and previous quit attempts. For this study, we focused on the variable for lifelong smokers - those who have smoked at least 100 cigarettes in their lifetime.

#### 2.2.7 General data

Associated questionnaires allow for collecting sociodemographic data, such as age, gender, marital status, education status, and work status. All sociodemographic variables and risk factor data (e.g., alcohol consumption, smoking, impulsivity, sleep habits) were collected at T1. This decision was based on the greater completeness of the data at this time point, as well as the fact that T1 assessments more accurately reflect patients’ typical behavior patterns prior to any stabilization or changes that may occur during hospitalization. No data was collected on the severity of BPD symptoms; therefore, this dimension was not included in the analysis.

### 2.3 Analysis

Descriptive statistics were conducted on sociodemographic characteristics and the prevalence of chronic conditions; quantitative variables are reported as means (with standard deviations) and percentages. Group comparisons for categorical variables were performed using Fisher’s exact test, selected due to small cell counts in several disease categories. P-values in **Table 1** were adjusted for multiple comparisons using the false discovery rate (FDR) method, applied separately within each of the three pairwise group comparisons.

**Table 1:** Comparison of disease prevalence among BPD, schizophrenia and control group with FDR adjusted p-values.

Disease	P-value (SCZ vs. BPD)	P-value (SCZ vs. Control)	P-value (BPD vs. Control)
Asthma	0.413	0.020*	0.181
Arthritis	0.413	0.331	0.882
Back problems	0.413	0.008*	0.130
Hypertension	0.811	0.943	0.822
Migraine	0.413	<0.001*	0.002*
Chronic bronchitis	0.070	0.051	0.908
Emphysema	0.102	1.000	0.169
COPD	0.156	1.000	0.408
Diabetes	0.070	0.943	0.181
Heart disease	0.811	0.573	0.411
Cancer	0.459	0.573	1.000
Gastrointestinal ulcers	0.004*	<0.001*	0.508
Stroke	0.613	0.943	0.882
Urinary incontinence	0.004*	0.021*	0.196
Bowel disorder	0.811	0.140	0.882
Dementia	0.225	0.706	0.882
Epilepsy	0.106	0.573	0.778

SCZ, Schizophrenia; BPD, Borderline Personality Disorder. P-values derived from Fisher’s exact test and adjusted using false discovery rate correction applied separately within each pairwise comparison. *p <0.05.

The primary analysis included logistic regression with odds ratios, adjusted for age and sex, to calculate the prevalence of multimorbidity among the three groups and ANOVA to compare the mean numbers of chronic conditions across the groups (based on the ESCC-2008 questionnaire). *Post-hoc* comparisons using Tukey’s HSD test were conducted to identify which groups differed when ANOVA indicated significant differences (p <0.05). Although the chronic disease count variable was right-skewed, ANOVA was retained for unadjusted group comparisons given the large sample size (n = 590) and its robustness to moderate deviations from normality. Adjusted analyses of chronic disease burden were performed using negative binomial regression.

A negative binomial regression model was used to assess the relationship between the chronic conditions score, treated as the count outcome, and diagnostic group, given evidence of overdispersion in the data under the Poisson model (dispersion = 1.75, p = 0.004). The model included the following covariates: alcohol use disorder score (AUDIT-10), tobacco use (defined as having smoked more than 100 cigarettes in a lifetime), sleep onset latency and nocturnal awakening time (SHQ), the five impulsivity subscales of the UPPS-P (negative urgency, positive urgency, lack of premeditation, lack of perseverance, and sensation-seeking), sex, age, education level, employment status, and income. Participants who responded “I don’t know” or “Refused to answer” to the tobacco question were excluded from this analysis, resulting in an analytic sample of 509 participants.

All analyses were conducted using complete-case analysis. The analytic sample size varied by model: logistic regression and ANOVA included 571 participants, whereas the negative binomial regression included 509 participants after excluding individuals with missing tobacco responses.

All statistical analyses were conducted using R software version 4.4.2, with a significance level established at α = 0.05 (two-tailed).

### 2.4 Power analysis

A power analysis conducted in G*Power indicated that a minimum sample size of 432 participants was required to detect an effect size of f=0.15 (small to medium) in an ANOVA, with 80% power and a type-I error rate of 5%. For Pearson correlations, a minimum of 82 participants is necessary to identify a correlation of 0.3 (in absolute value) with 80% power and a type-I error rate of 5%. Since our sample consisted of 590 participants (158 + 283 + 149), this study was sufficiently powered to conduct all planned analyses.

## 3 Results

### 3.1 Sample characteristics

The BPD group was 70.9% female, the schizophrenia group 78.4% male, and the control group 53% female (see **Table 2**). Overall, females represented 41.2% of the total sample across the three groups. Regarding marital status, both the BPD (86.5%) and schizophrenia patients (90.8%) had a significantly higher proportion of individuals who had never been legally married compared to the control group (69.6%). In terms of education, a higher proportion of individuals in the BPD group (46.9%) and the schizophrenia group (45%) had not completed high school, in contrast to just 6.1% in the control group. Conversely, the control group had a higher percentage of individuals who had completed a bachelor’s degree or higher. Finally, concerning employment status, patients with schizophrenia exhibited the highest unemployment rate (85.2%), followed by the BPD group at 61.9%. The control group had a lower unemployment rate of 43.95% compared to the other two groups, but a higher rate than the general average of 6.9% in Montreal. This group was composed primarily of participants from Hochelaga-Maisonneuve, a borough of Montreal with a more socioeconomically disadvantaged profile (24).

**Table 2:** Sociodemographic characteristics by group.

Variable	Control	Schizophrenia	BPD	P test
N	149	283	158	
Age (mean [SD])	41.16 (16.49)	39.14 (13.59)	32.42 (12.08)	<0.001
Sex = male (N [%])	79 (53.0)	222 (78.4)	46 (29.1)	<0.001
Category of age (N [%])				<0.001
<20 years	5 (3.4)	8 (2.8)	13 (8.2)	
20–29 years	42 (28.4)	78 (27.6)	65 (41.1)	
30–39 years	32 (21.6)	76 (26.9)	44 (27.8)	
40–49 years	18 (12.2)	49 (17.3)	19 (12.0)	
50–59 years	27 (18.2)	44 (15.5)	11 (7.0)	
60–69 years	13 (8.8)	25 (8.8)	5 (3.2)	
>=70 years	11 (7.4)	3 (1.1)	1 (0.6)	
Ethnicity (%)				<0.001
White	123 (83.1)	176 (62.4)	146 (93.0)	
South Asian	2 (1.4)	0 (0.0)	0 (0.0)	
Chinese	2 (1.4)	0 (0.0)	0 (0.0)	
Black	7 (4.7)	68 (24.1)	4 (2.5)	
Latino	4 (2.7)	6 (2.1)	1 (0.6)	
Southeast Asian	1 (0.7)	0 (0.0)	0 (0.0)	
West Asian	0 (0.0)	1 (0.4)	1 (0.6)	
Indigenous	1 (0.7)	4 (1.4)	0 (0.0)	
Others	2 (1.4)	0 (0.0)	0 (0.0)	
Arabic/North African/Middle East	3 (2.0)	17 (6.0)	2 (1.3)	
East Asian	0 (0.0)	2 (0.7)	0 (0.0)	
Caribbean (except Haiti)	0 (0.0)	2 (0.7)	0 (0.0)	
Mixed origin	3 (2.0)	6 (2.1)	3 (1.9)	
Marital status (%)				<0.001
Never legally married	103 (69.6)	256 (90.8)	134 (86.5)	
Legally married (and not separated)	32 (21.6)	5 (1.8)	5 (3.2)	
Separated, but still legally married	2 (1.4)	3 (1.1)	3 (1.9)	
Divorced	10 (6.8)	18 (6.4)	11 (7.1)	
Widowed	1 (0.7)	0 (0.0)	2 (1.3)	
Common law = No (%)	108 (73.5)	267 (95.4)	131 (85.1)	<0.001
Highest grade completed (%)				<0.001
Secondary II or less (Grade 8 or less)	3 (2.0)	53 (18.9)	22 (14.1)	
Secondary III or IV (Grade 9 or 10)	7 (4.7)	68 (24.3)	37 (23.7)	
Secondary V (Grade 11)	138 (93.2)	159 (56.8)	97 (62.2)	
Highest level of education (%)				<0.001
Lower than a high school diploma or its equivalent	9 (6.1)	117 (45.0)	67 (46.9)	
High school diploma or a high school equivalency certificate	19 (12.9)	69 (26.5)	26 (18.2)	
Certificate or diploma from a trade school	10 (6.8)	23 (8.8)	14 (9.8)	
Certificate or diploma from a college, CEGEP, or another non-university institution (other than trade certificates or diplomas)	20 (13.6)	27 (10.4)	17 (11.9)	
Certificate or diploma below the bachelor’s degree level	9 (6.1)	12 (4.6)	10 (7.0)	
Bachelor’s degree (e.g., B.A., B.Sc., LL.B.)	43 (29.3)	9 (3.5)	4 (2.8)	
Certificate, diploma, or degree above the bachelor’s degree level	37 (25.2)	3 (1.2)	5 (3.5)	
Currently employed= No (%)	65 (43.9)	241 (85.2)	96 (61.9)	<0.001
Household composition (lives with) (%)				<0.001
A - Partner	22 (40.7)	12 (4.2)	27 (17.3)	
B - Parent(s)	4 (7.4)	75 (26.5)	28 (17.9)	
C - Children	1 (1.9)	4 (1.4)	10 (6.4)	
D - Other family members	4 (7.4)	22 (7.8)	10 (6.4)	
E - Other patients	0 (0.0)	29 (10.2)	5 (3.2)	
F - Other people	8 (14.8)	26 (9.2)	16 (10.3)	
G - Alone	15 (27.8)	115 (40.6)	60 (38.5)	
Living situation (%)				<0.001
Supervised apartment (shared or not)	0 (0.0)	11 (3.9)	2 (1.3)	
Other, specify below	2 (1.4)	13 (4.6)	3 (1.9)	
Hospital or psychiatric unit	0 (0.0)	0 (0.0)	2 (1.3)	
Group home or residence with 10 or more users (Pavilion)	0 (0.0)	17 (6.0)	0 (0.0)	
Group home or residence with 9 or fewer users	0 (0.0)	16 (5.7)	3 (1.9)	
Judicial hospital (Philippe Pinel Institute)	0 (0.0)	0 (0.0)	2 (1.3)	
Independent living in HLM or non-profit housing (social housing) with services, support, or assistance	0 (0.0)	3 (1.1)	2 (1.3)	
Independent living in HLM or non-profit housing (social housing) without services, support, or assistance	4 (2.7)	15 (5.3)	3 (1.9)	
Cooperative housing (coop)	0 (0.0)	3 (1.1)	1 (0.6)	
Housing, private house, or condo	142 (95.9)	182 (64.5)	133 (85.3)	
Boarding house with services, support, or assistance	0 (0.0)	4 (1.4)	0 (0.0)	
Boarding house without services, support, or assistance	0 (0.0)	4 (1.4)	1 (0.6)	
Foster home or RTF (Residential Treatment Facility)	0 (0.0)	1 (0.4)	0 (0.0)	
Homeless	0 (0.0)	9 (3.2)	3 (1.9)	

### 3.2 Comorbidities and multimorbidity

A higher proportion of individuals in the BPD group reported issues such as back problems, migraines, chronic bronchitis, gastrointestinal ulcers, and bowel disorders (see **Table 3**). In contrast, the schizophrenia group showed a higher proportion of individuals with emphysema, chronic obstructive pulmonary disease (COPD), diabetes, and urinary incontinence.

**Table 3:** Prevalence of disease by group.

Variable	Control	Schizophrenia	BPD
N	**149**	**283**	**158**
Asthma	13 (9.4%)	49 (14.0%)	41 (22.0%)
Arthritis	9 (6.0%)	36 (9.5%)	25 (11.3%)
Back problems	34 (22.8%)	88 (28.6%)	69 (40.7%)
Hypertension	16 (10.7%)	43 (12.1%)	22 (9.3%)
Migraine	25 (16.8%)	69 (21.6%)	74 (44.0%)
Chronic bronchitis	3 (2.0%)	32 (8.1%)	21 (8.7%)
Emphysema	0 (0.0%)	19 (3.3%)	0 (0.0%)
COPD	0 (0.0%)	18 (2.9%)	1 (0.7%)
Diabetes	7 (4.7%)	44 (12.5%)	17 (6.0%)
Heart disease	6 (4.0%)	24 (5.1%)	11 (2.0%)
Cancer	3 (2.0%)	2 (0.7%)	1 (0.7%)
Gastrointestinal ulcers	0 (0.0%)	29 (7.0%)	23 (10.0%)
Stroke	2 (1.3%)	18 (2.9%)	2 (2.0%)
Urinary incontinence	2 (1.3%)	37 (9.9%)	21 (8.7%)
Bowel disorder	7 (4.7%)	26 (5.9%)	25 (11.3%)
Dementia	0 (0.0%)	16 (2.2%)	2 (1.3%)
Epilepsy	1 (0.7%)	20 (3.7%)	12 (2.7%)

The bold values are the number of participants in each group.

Among patients with BPD, the most prevalent conditions were migraine (44.0%), back issues (40.7%), and asthma (22.0%). Similarly, individuals with schizophrenia reported back issues (28.6%), migraines (21.6%), and asthma (14.0%) as the most common conditions. For the control group, the most frequent comorbidities were back issues (22.8%), migraine (16.8%), and hypertension (10.7%).

For most diseases, no significant differences in prevalence were observed between the groups after FDR correction. However, several findings are noteworthy. A significant difference between individuals with schizophrenia and those with BPD was observed for gastrointestinal ulcers and urinary incontinence. When comparing individuals with schizophrenia to the control group, significant differences were found for asthma, back problems, migraine, gastrointestinal ulcers, and urinary incontinence. Finally, migraine was the only condition that showed a significant difference between the BPD and control groups (see **Table 1**). Overall, these findings suggest that significant differences in physical comorbidities were limited after correction for multiple testing.

**Tables 4**, **5** illustrate the distribution of participants based on the number of chronic diseases and multimorbidity. After adjustment for age and sex, BPD presented the highest proportion of multimorbidity at 49.4%, with an odds ratio of 7.21 (95% CI: 4.03-13.3, p <0.001) when compared to the control group. Schizophrenia also showed a higher proportion of multimorbidity than the control group, with an odds ratio of 3.51 (95% CI: 2.10-6.02, p <0.001).

**Table 4:** Distribution of chronic disease categories across groups.

Group	0 Diseases (%)	1 Disease (%)	Multimorbidity 2+ diseases (%)	Missing data (%)
Control (149)	49.7	30.2	20.1	0.0
Schizophrenia (283)	38.2	21.6	36.4	3.9
BPD (158)	22.8	22.8	49.4	5.1

**Table 5:** Odds ratios for having multimorbidity, adjusted for age and sex.

Comparison	Odds Ratio	95% CI (Lower - Upper)	P-value
Schizophrenia vs. Control	3.51	2.10 - 6.02	<0.001 ***
BPD vs. Control	7.21	4.03 - 13.3	<0.001 ***

The mean number of chronic diseases was 0.86 (SD = 1.17) in the control group, 1.49 (SD = 2.04) in the schizophrenia group, and 1.91 (SD = 1.62) in the BPD group. An analysis of variance (ANOVA) was conducted to compare the mean number of chronic diseases across the three groups. The results revealed a significant difference between groups, F (2, 568) = 11.52, p <.001.

Tukey’s Honest Significant Difference (HSD) *post-hoc* tests were conducted to examine these differences further. Pairwise comparisons indicated that individuals with schizophrenia significantly differed from the control group (mean difference = 0.623, p = 0.001). Similarly, individuals with BPD had significantly more chronic diseases than the control group (mean difference = 0.948, p <0.001). However, no significant difference was found between the schizophrenia and BPD groups (mean difference = 0.324, p = 0.162).

### 3.3 Factors associated with chronic disease burden

The negative binomial regression model was statistically significant overall (Please see **Table 6**). Individuals with schizophrenia had a 63% higher chronic disease burden than controls after adjustment for all covariates (IRR = 1.63, 95% CI: 1.17-2.28, p = 0.004), while individuals with BPD had a 70% higher chronic disease burden than controls (IRR = 1.70, 95% CI: 1.16-2.48, p = 0.006). Longer sleep latency was significantly associated with a higher chronic disease burden (IRR = 1.003, p <0.001), as was negative urgency (IRR = 1.07, p < 0.001). Being a non-smoker was significantly associated with a lower chronic disease burden (IRR = 0.735, p = 0.009), while alcohol use was not significantly associated with the outcome (p = 0.719). Male gender was also linked to a lower chronic disease burden (IRR = 0.764, p = 0.019). Age showed the strongest association (IRR = 1.02, p < 0.001). Education level, employment status, and income were not significant predictors, with the exception of having a high school diploma compared with less than a high school education, which was associated with a lower chronic disease burden (IRR = 0.741, p = 0.030).

**Table 6:** Negative binomial regression analysis of chronic disease burden.

Variable	IRR	95% CI	P-value
(Intercept)	0.250	0.120-0.516	<0.001***
Group (ref: control)			
Schizophrenia	1.63	1.17-2.28	0.004**
BPD	1.70	1.16-2.48	0.006**
Alcohol use	0.997	0.983-1.01	0.719
Non-Smoker	0.735	0.582-0.927	0.009**
Sleep Latency	1.003	1.002-1.005	<0.001***
Awake Time	1.000	0.999-1.001	0.844
Negative Urgency	1.07	1.03-1.12	<0.001***
Positive Urgency	0.985	0.943-1.03	0.499
Lack of Premeditation	0.982	0.938-1.03	0.428
Lack of Perseverance	1.03	0.986-1.07	0.187
Sensation-Seeking	0.985	0.950-1.02	0.407
Male (Ref: Female)	0.764	0.612-0.954	0.019*
Age	1.02	1.02-1.03	<0.001***
High School Diploma (Ref: Below High School)	0.741	0.564-0.971	0.030*
Trade Certificate	1.02	0.724-1.44	0.905
College Diploma	0.764	0.525-1.10	0.151
Below-Bachelor University	0.998	0.647-1.52	0.994
Bachelor’s Degree	1.26	0.826-1.91	0.271
Graduate Degree	0.724	0.431-1.20	0.219
Unemployed (Ref: Employed)	1.08	0.836-1.40	0.544
Income	1.00	1.00-1.00	0.388

IRR, Incidence Rate Ratio; Ref, Reference category. Significance: *p<0.05, **p<0.01, ***p<0.001.

To test whether sex moderated the association between diagnostic group and chronic disease burden, an interaction term between group and sex was added to the model. Neither interaction was statistically significant (schizophrenia × male: IRR = 1.09, p = 0.761; BPD × male: IRR = 1.58, p = 0.118), indicating that the association between diagnosis and chronic disease burden did not differ by sex.

## 4 Discussion

This study uniquely provides a direct comparison of three groups: individuals with BPD, those with schizophrenia, and healthy controls. Our primary findings include: (a) a significantly higher rate of multimorbidity among BPD patients; (b) significant differences in the average number of chronic conditions across the three groups; and (c) the identification of age, sleep parameters, impulsivity, sex, and smoking status as key correlates of comorbidity.

Migraine emerged as the condition with the most statistically significant difference after FDR correction, both between BPD and control group, as well as between schizophrenia and control group. Migraine is often associated with high levels of neuroticism, a personality trait commonly observed in individuals with BPD. Neuroticism is associated with increased vulnerability to negative affect and stress. Among patients with refractory daily headaches, 26% present with a personality disorder (25), and this population tends to report more pervasive symptoms, medication overuse, and poorer responses to treatment (25). Evidence regarding migraine in schizophrenia remains mixed: some studies suggest a positive association, indicating that schizophrenia may be linked to an increased risk of migraine (26), while others report an inverse relationship, with individuals with schizophrenia exhibiting lower rates of migraine than the general population (27).

In addition to these findings, schizophrenia showed a significantly higher prevalence of pulmonary emphysema and chronic bronchitis compared to the BPD group, as well as higher rates of chronic bronchitis and asthma compared to controls. Although elevated rates of asthma, COPD, pneumonia, and tuberculosis have been reported in individuals with schizophrenia (28), and increased respiratory disease has also been noted in this population (29), none of these differences remained statistically significant after correction for multiple comparisons. These findings should therefore be interpreted as non-significant group-level variations.

We observed that longer sleep latency was significantly associated with higher multimorbidity, although the cross-sectional design does not allow conclusions regarding causality. Sleep latency, often used as a proxy for insomnia (30), is highly prevalent in both BPD and schizophrenia. Sixty-three percent of individuals with BPD report some form of sleep difficulty (31), while estimates for those with schizophrenia range from 30% to 80% (32). Initial insomnia, characterized by prolonged sleep onset, has been associated with metabolic issues (33). This association is supported by findings from a study in a Canadian First Nation population, where longer sleep onset latency was correlated with several health and environmental factors, including older age, exposure to indoor smoke, chronic pain, anxiety, and heart disease (34). Elevated levels of inflammatory markers such as CRP, TNF-α, and IL-10 are also reported in individuals with insomnia (35). These inflammatory responses are implicated in various chronic conditions, including cardiovascular disease (36). Another proposed mechanism suggests that sleep disorders contribute to dysregulation of the HPA axis, resulting in heightened stress responses, increased glucocorticoid secretion, insulin resistance, and the onset of metabolic diseases (37). Although the effect sizes observed for sleep latency were modest, their cumulative impact may be clinically meaningful given the high prevalence of sleep disturbances and emotion dysregulation among individuals with BPD and schizophrenia.

The impulsivity dimension of negative urgency, defined as the tendency to act impulsively under distress, was also associated with a higher burden of chronic disease, although this association remains correlational given the cross-sectional design. It is also possible that chronic disease burden, pain, or functional limitations may contribute to emotional dysregulation and impulsive responses, reflecting potential bidirectional associations. Affective instability, often present in individuals with high negative urgency, has been associated with maladaptive coping strategies such as binge eating, resulting in weight gain (38, 39). This is consistent with evidence that BPD, as an impulse control disorder, is particularly associated with binge-eating disorder and obesity (40), likely through similar affective dysregulation pathways implicated in negative urgency. Individuals who are overweight or obese often display higher impulsivity, stronger sensation-seeking behaviors, and lower levels of self-discipline (41). In addition, among patients with unipolar and bipolar depression, those with a history of non-suicidal self-injury, a behavior linked to impulsivity, showed elevated inflammatory markers compared to those without such a history (42). A longitudinal study further found that impulsivity predicted a higher incidence of type 2 diabetes over an eight-year period (42). In contrast, other impulsivity traits in our study, such as positive urgency and sensation-seeking, were not significantly associated with chronic disease burden. Negative urgency was the only impulsivity dimension that remained significant in the adjusted model, indicating that this association may be more specific to impulsive behaviors occurring in the context of negative emotions.

Our findings are consistent with well-established evidence that tobacco use is associated with an increased risk of chronic diseases and multimorbidity (43). However, given the cross-sectional design, the direction of this association cannot be established, as smoking may also represent a coping strategy in response to psychiatric symptoms, stress, or the burden of chronic illness. Smoking is responsible for approximately 8 million deaths annually (44), many of which are premature and preventable (45). The prevalence of smoking is notably higher among individuals with psychiatric disorders (46), which may further contribute to their risk for chronic disease.

In our findings, age emerged as the strongest correlate of chronic disease burden, while male sex was associated with a lower burden. This is consistent with existing literature showing that multimorbidity increases with age and is generally higher in women (47). Similarly, a study found higher rates of multimorbidity in adults over the age of 60, particularly among individuals with personality disorders (48). Furthermore, women tend to experience a greater burden of chronic disease than men, even in younger age groups (49), which is consistent with the lower burden observed among males in our sample. However, the association with male sex should be interpreted in the context of the substantial gender imbalance across groups, which may have partially influenced group comparisons.

Education, employment, and income did not emerge as significant independent predictors in our analysis. This contrasts with previous research, which has consistently shown that lower education levels are associated with increased multimorbidity (50, 51). Other studies have reported that broader socioeconomic disadvantage is linked to a greater burden of chronic disease (52). One possible reason for this discrepancy is the socioeconomic profile of our study population. All participants were recruited from a socioeconomically disadvantaged area of Montreal, which has higher unemployment rates, lower median household income and educational attainment relative to the rest of the city. This may have reduced our ability to detect associations between these socioeconomic variables and chronic disease burden. It is also possible that the effects of socioeconomic status are mediated through behavioral factors, such as smoking, impulsivity, or poor sleep, which were accounted for separately in our model. By modeling these behaviors directly, we may have reduced the observable direct effect of education or income on health outcomes. In our sample, only participants whose highest level of education was below high school showed a trend toward higher disease burden, suggesting that educational effects may only become evident below a certain threshold. Regarding income, although it is typically considered protective (14), the lack of association in our data may reflect contextual factors such as informal income reporting, variability in cost of living, or access to public services that buffer financial disadvantage. These results suggest the need for future studies to better disentangle the direct and indirect pathways through which socioeconomic factors contribute to multimorbidity, especially in clinical populations with complex behavioral and environmental risk profiles.

One of the primary strengths of this study lies in its focus on a relatively understudied area, namely the chronic disease burden among individuals with severe psychiatric conditions. Another strength is the comparison of two distinct psychiatric disorders, which nonetheless share numerous associated characteristics for chronic disease and multimorbidity. In addition, the large sample size and the assessment of behavioral characteristics, including tobacco use, sleep habits, and impulsivity, provided an opportunity to identify potentially modifiable correlates of physical health burden. Notably, negative urgency emerged as a significant predictor of chronic disease burden, suggesting that impulsivity-related factors may play an important role in physical health outcomes among individuals with severe mental illness.

Several limitations should be considered when interpreting these findings. Both clinical groups were recruited from a psychiatric emergency setting, which likely represents individuals with more severe symptoms and greater functional impairment than community samples of either disorder. For BPD, hospitalization is relatively less common, suggesting that those admitted may represent a more complex subgroup. For schizophrenia, emergency presentations may overrepresent individuals with poorer medication adherence or fewer community supports. The gender imbalance across groups, with the BPD group being predominantly female and the schizophrenia group predominantly male, may have partially confounded group comparisons, particularly as sex and age were not adjusted for in the ANOVA. False discovery rate correction was applied to disease-specific prevalence comparisons in **Table 1**, and several findings that appeared significant before correction did not survive adjustment and should be interpreted cautiously. Furthermore, the cross-sectional design does not allow conclusions regarding causality, self-reported chronic conditions may have introduced reporting bias, and the simple count measure does not account for differences in disease severity or clinical impact. In addition, differential healthcare access and utilization across groups may have influenced the reported prevalence of conditions, as some diagnoses may reflect differences in diagnostic access rather than true differences in disease burden. Objective medical records or healthcare utilization data were not available to validate self-reported conditions, which should be considered when interpreting disease-specific findings.

This study adds to growing evidence that the separation between mental and physical health is no longer tenable. After adjustment for demographic and behavioral characteristics, individuals with BPD and schizophrenia continued to show a higher burden of chronic disease compared to controls, with age and negative urgency identified as key correlates. These findings suggest the importance of addressing emotion-driven impulsivity to help manage multimorbidity, consistent with previous evidence supporting mindfulness-based interventions (53). Although psychotherapeutic approaches are often prioritized in mental health care, physical health is frequently overlooked, which may contribute to worse health outcomes (54, 55). Interventions such as smoking cessation and sleep improvement may represent promising components to reduce chronic disease burden. Translating these findings into clinical practice requires considering the specific challenges of intervening in individuals with BPD, whose emotional instability and impulsivity may affect adherence to conventional health promotion programs (56). Adapted interventions may be needed, including combining cognitive behavior therapy for insomnia with emotion regulation training, and using mindfulness-based approaches, which may reduce negative urgency and improve self-regulation (57, 58). Future longitudinal cohort studies are needed to examine whether changes in factors such as sleep latency, impulsivity, and smoking are associated with the development of chronic diseases over time among individuals with BPD and schizophrenia. Beyond these priorities, future research may also benefit from examining specific conditions that are frequently overlooked in this population, including chronic pain and polyendocrine metabolic ovarian syndrome (PMOS), given their known associations with both psychiatric disorders and metabolic dysregulation.

## Data Availability

The original contributions presented in the study are included in the article/supplementary material. Further inquiries can be directed to the corresponding author.
